# Supplementary material for: Immunotherapies targeting the oncogenic fusion gene CLDN18-ARHGAP in gastric cancer
Source: EMBO Mol Med. 2024 Aug 20;16(9):2170–87. doi: 10.1038/s44321-024-00120-3 (PMC11393071; doi:10.1038/s44321-024-00120-3)
Supplement: Supplementary file 1 — Appendix [file 44321_2024_120_MOESM1_ESM.pdf]

Appendix

Immunotherapies targeting the oncogenic fusion gene CLDN18-ARHGAP in gastric cancer

Table of contents

Appendix Table. S1 •••••••••••••••••••••••••••••••••• 2

Appendix Table. S2 •••••••••••••••••••••••••••••••••• 3

Appendix Fig. S1 •••••••••••••••••••••••••••••••••••• 4

Appendix Fig. S2 •••••••••••••••••••••••••••••••••••• 5

Appendix Fig. S3 •••••••••••••••••••••••••••••••••••• 5

Appendix Fig. S4 •••••••••••••••••••••••••••••••••••• 7

Appendix Fig. S5 •••••••••••••••••••••••••••••••••••• 8

Appendix Fig. S6 •••••••••••••••••••••••••••••••••••• 9

Appendix Tables

Appendix Table. S1. Neoantigens derived from CLDN18/exon5-ARHGAP26/exon10

Name	Gene	Sequence	Rank
	CLDN18(e5)-ARHGAP26(e10)	YDGGARTEDEGEDESVILKS	
P5	CLDN18(e5)-ARHGAP26(e10)	EGEDESVILK	4.7
P6	CLDN18(e5)-ARHGAP26(e10)	DEGEDESVILK	11.3
	CLDN18	YDGGARTEDEVQSYPSKHDY	
WT5-1	CLDN18	EVQSYPSKHD	75.0
WT6-1	CLDN18	DEVQSYPSKHD	85.0
	ARHGAP26	PFDQKSGGKGGEDESVILKS	
WT5-2	ARHGAP26	GGEDESVILK	2.0
WT6-2	ARHGAP26	KGGEDESVILK	2.4

Amino acid from CLDN18 and ARHGAP26 gene were indicated with green and red letters respectively. P5 and P6 were versicolor sequences, representing neoantigens from CLDN18/exon5-ARHGAP26/exon10; WT5-1 and WT6-1 were green sequences, representing wildtype peptides from CLDN18 gene; WT5-2 and WT6-2 were red sequences, representing wildtype peptides from ARHGAP26 gene.

Appendix Table. S2. Neoantigens derived from CLDN18/exon5-ARHGAP6/exon2

Name	Gene	Sequence	Rank
	CLDN18(e5)-ARHGAP6(e2)	YDGGARTEDEGDFTWNSMSG	
P7	CLDN18(e5)-ARHGAP6(e2)	RTEDEGDFTW	9.6
P8	CLDN18(e5)-ARHGAP6(e2)	DEGDFTWNSM	39.5
	CLDN18	YDGGARTEDEVQSYPSKHDY	
WT7-1	CLDN18	RTEDEVQSYP	13.2
WT8-1	CLDN18	DEVQSYPSKH	34.0
	ARHGAP6	GHPYVVWKSEGDFTWNSMSG	
WT7-2	ARHGAP6	VWKSEGDFTW	17.3
WT8-2	ARHGAP6	SEGDFTWNSM	24.7

Amino acid from CLDN18 and ARHGAP6 gene were indicated with green and red letters respectively. P7 and P8 were versicolor sequences, representing neoantigens from CLDN18/exon5-ARHGAP6/exon2; WT7-1 and WT8-1 were green sequences, representing wildtype peptides from CLDN18 gene; WT7-2 and WT8-2 were red sequences, representing wildtype peptides from ARHGAP6 gene.

Appendix Figures

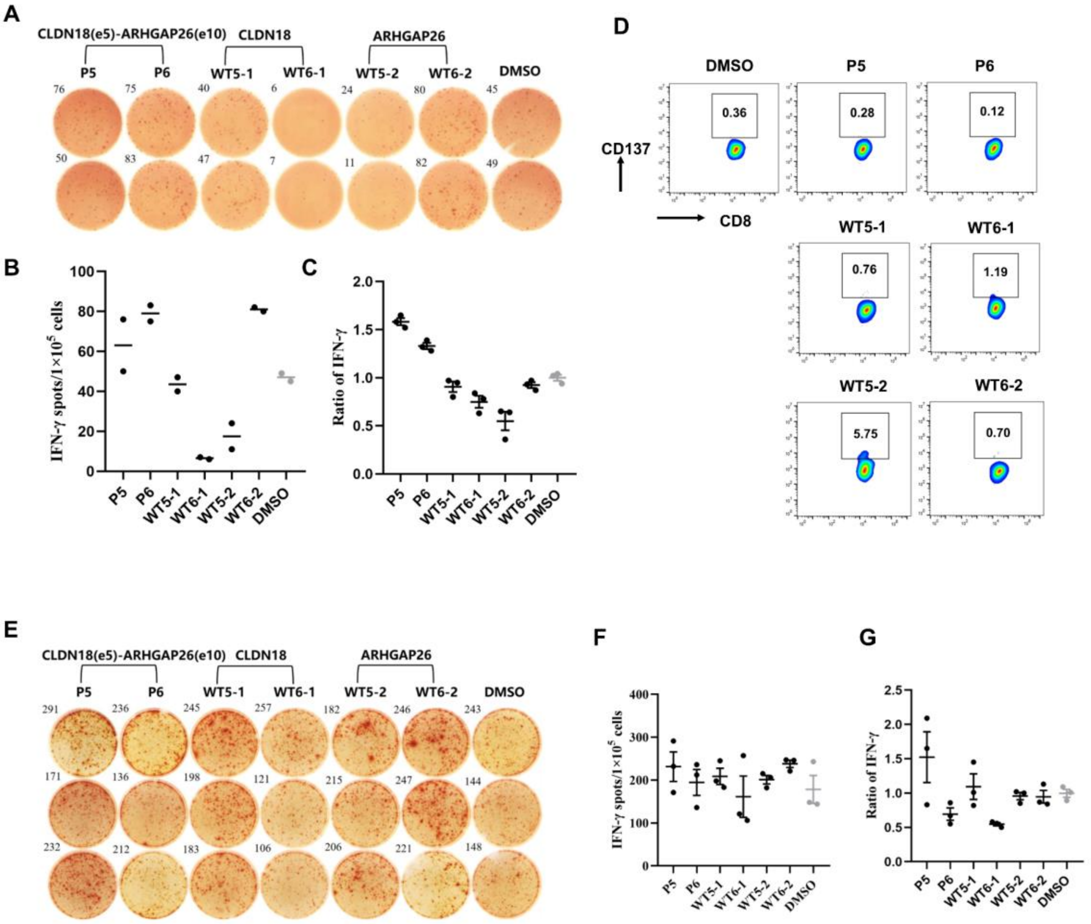

Appendix Fig. S1. Identification of neoantigens from CLDN18/exon5-ARHGAP26/exon10 fusion in autologous and donor's T cells. Autologous PBMCs from PC GC patient with HLA-A*11:01 typing were isolated and stimulated with CLDN18(e5)-ARHGAP26(e10)-derived peptides, CLDN18-derived WT peptides or ARHGAP26-derived WT peptides every 3 days in the presence of IL-2, and T cell response to each peptide were analyzed on day 10 by **(A)** IFN-γ ELISPOT assays, **(B)** quantification of IFN-γ spots and **(C)** ratio of IFN-γ secretion shown in A, and **(D)** detection of CD137 expression on $CD8^+$ T cells via flow cytometry. T cells from healthy donors were isolated and stimulated with APCs loading candidate peptides every 7 days in the presence of IL-2, and T cell response to each peptide were analyzed on day 15 by **(E)** IFN-γ ELISPOT assays, **(F)** quantification of IFN-γ spots and **(G)** ratio of IFN-γ secretion shown in E. The increase of IFN-γ more than 2 folds was considered to be different.

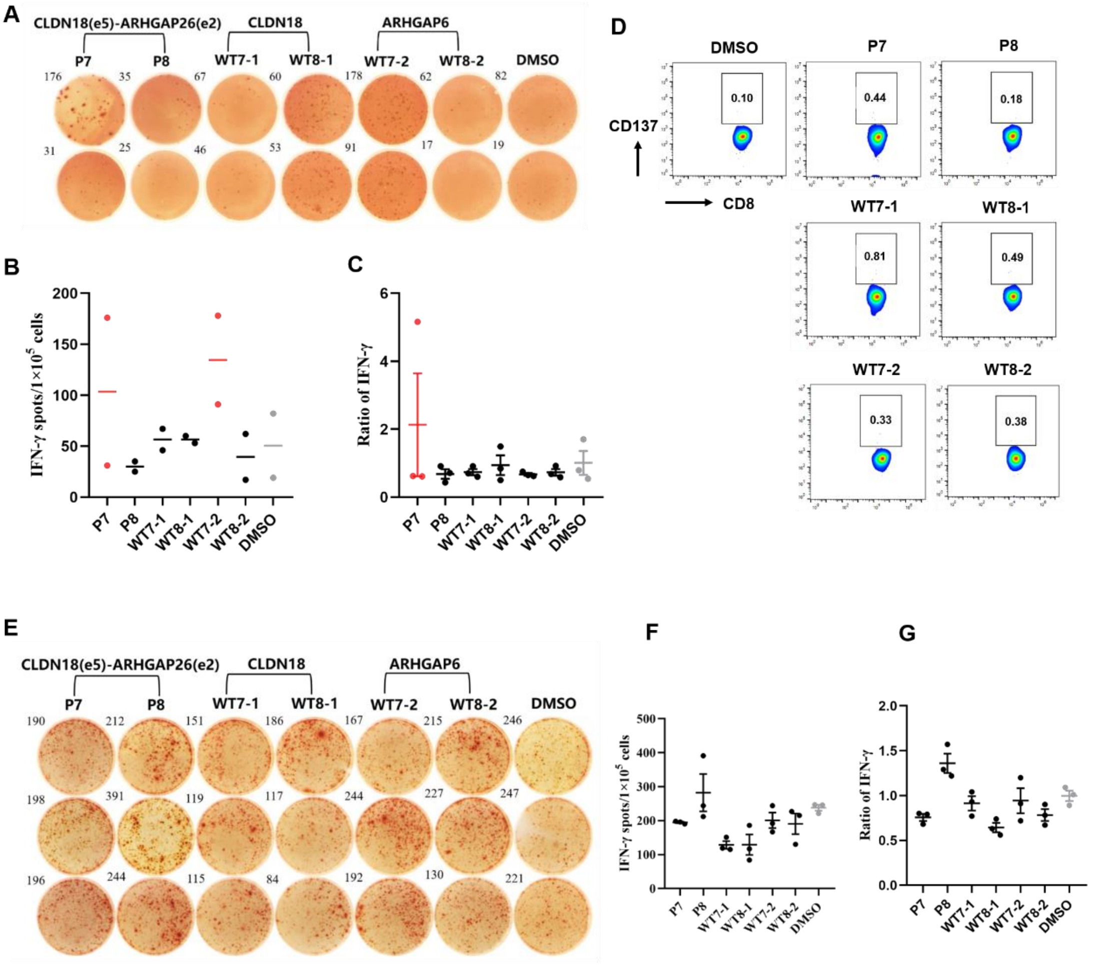

Appendix Fig. S2. Identification of neoantigens from CLDN18/exon5-ARHGAP26/exon2 fusion in autologous and donor's T cells. Autologous PBMCs from PC GC patient with HLA-A*11:01 typing were isolated and stimulated with CLDN18(e5)-ARHGAP26(e2)-derived peptides, CLDN18-derived WT peptides or ARHGAP26-derived WT peptides every 3 days in the presence of IL-2, and T cell response to each peptide were analyzed on day 10 by **(A)** IFN-γ ELISPOT assays, **(B)** quantification of IFN-γ spots and **(C)** ratio of IFN-γ secretion shown in A, and **(D)** detection of CD137 expression on $CD8^+$ T cells via flow cytometry. T cells from healthy donors were isolated and stimulated with APCs loading candidate peptides every 7 days in the presence of IL-2, and T cell response to each peptide were analyzed on day 15 by **(E)** IFN-γ ELISPOT assays, **(F)** quantification of IFN-γ spots and **(G)** ratio of IFN-γ secretion shown in E. The increase of IFN-γ more than 2 folds was considered to be different.

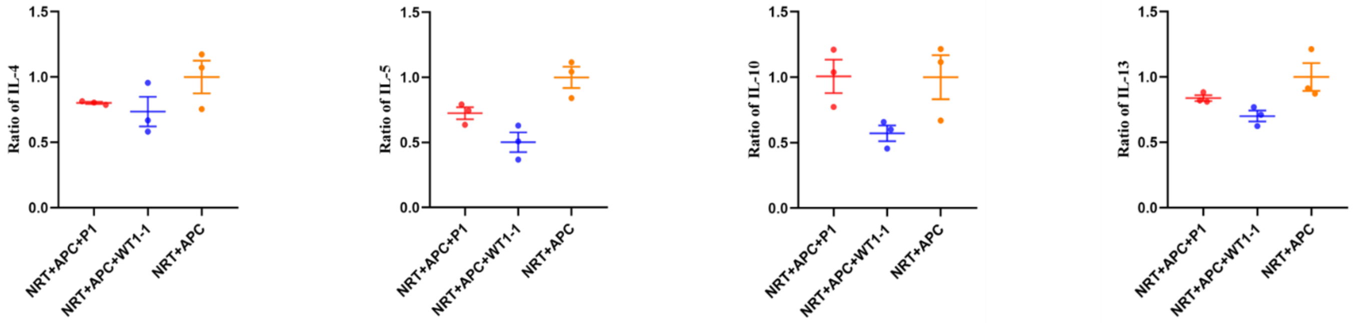

Appendix Fig. S3. Immunosuppressive cytokines including IL-4, IL-5, IL-10 and IL-13 from the culture of donor T cells stimulated with IL-2 and APCs loading P1 or WT peptides were detected by CBA. The increase of IL-4, IL-5, IL-10 or IL-13 more than 2 folds was considered to be different.

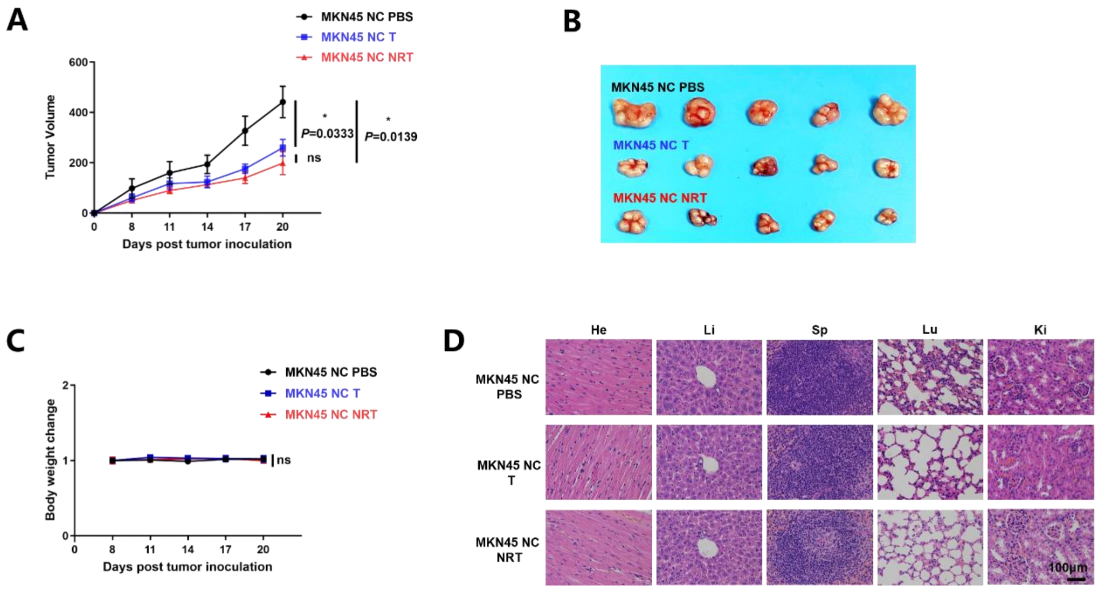

Appendix Fig. S4. NRT cells targeting CLDN18-ARHGAP fusion have no specific cytotoxicity on GC model without CLDN18-ARHGAP fusion. Balb/c nude mice (n=5 per group) were injected s.c. with 10^7 MKN45 cells and treated i.v. with T or P1-induced NRT cells twice every 6 days. **(A)** Tumor volumes of mice treated with T or NRT cells (n=5 per group). **(B)** Mice were scarified at treatment endpoint and tumors were removed for analysis. **(C)** Body weight of mice treated with T or NRT cells (n=5 per group). **(D)** Safety evaluation of T or NRT cells in mouse organs including heart, liver, spleen, lung and kidney shown by hematoxylin and eosin (H&E) staining (Scale bars, 100 μm). Data with error bars are shown as mean ± SEM. *$P < 0.05$, **$P < 0.01$, ***$P < 0.001$, ****$P < 0.0001$.

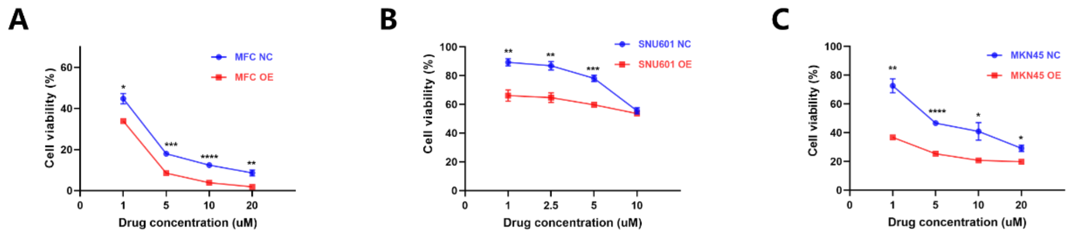

Appendix Fig. S5. Sensitivity of GC cells to the PI3K inhibition. Sensitivity of (A) SNU601 NC/OE, (B) MKN45 NC/OE and (C) MFC NC/OE cell to the PI3K inhibitor Pictilisib (48h). Data with error bars are shown as mean ± SEM. *$P < 0.05$, **$P < 0.01$, ***$P < 0.001$, ****$P < 0.0001$.

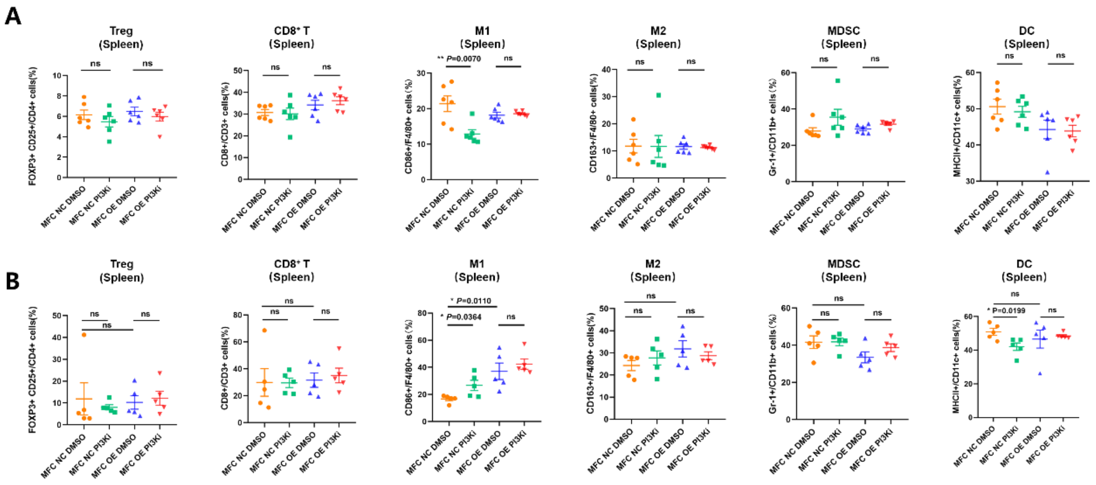

Appendix Fig. S6. (A) The proportions of FOXP3$^+$ CD25$^+$ / CD4$^+$ cells, CD8$^+$ / CD3$^+$ cells, CD86$^+$ / F4/80$^+$ cells, CD163$^+$ / F4/80$^+$ cells, Gr-1$^+$/CD11b$^+$ cells and MHC-II$^+$ / CD11c$^+$ cells in spleen of MFC OE/NC subcutaneously challenged 615-line mice were determined by flow cytometry. **(B)** The proportions of FOXP3$^+$ CD25$^+$ / CD4$^+$ cells, CD8$^+$ / CD3$^+$ cells, CD86$^+$ / F4/80$^+$ cells, CD163$^+$ / F4/80$^+$ cells, Gr-1$^+$/CD11b$^+$ cells and MHC-II$^+$ / CD11c$^+$ cells in spleen of MFC OE/NC abdominally challenged 615-line mice were determined by flow cytometry. Data with error bars are shown as mean ± SEM. *$P < 0.05$, **$P < 0.01$, ***$P < 0.001$, ****$P < 0.0001$.
