# Supplementary figures and images for: Immunotherapies targeting the oncogenic fusion gene CLDN18-ARHGAP in gastric cancer
Source: EMBO Mol Med. 2024 Aug 20;16(9):2170–87. doi: 10.1038/s44321-024-00120-3 (PMC11393071; doi:10.1038/s44321-024-00120-3)

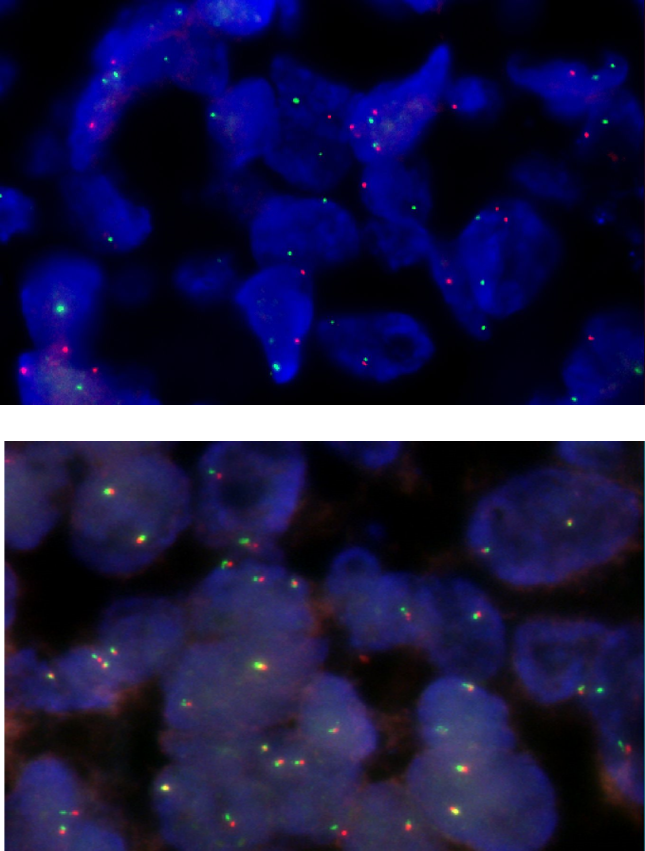

Supplement: Supplementary file 3 — Source data Fig. 1 [file 44321_2024_120_MOESM3_ESM.zip › Figure1 Source Data/Fig. 1C/Fig. 1C.tif]

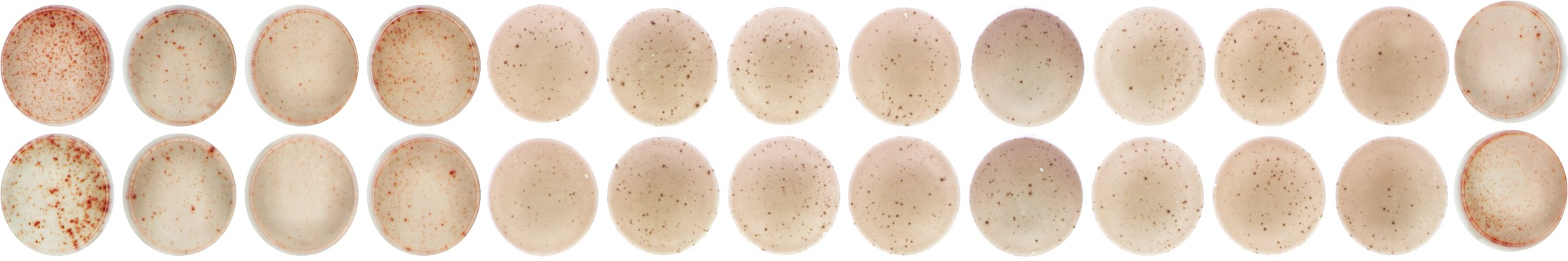

Supplement: Supplementary file 4 — Source data Fig. 2 [file 44321_2024_120_MOESM4_ESM.zip › Figure2 Source Data/Fig. 2A/Fig. 2A.tif]

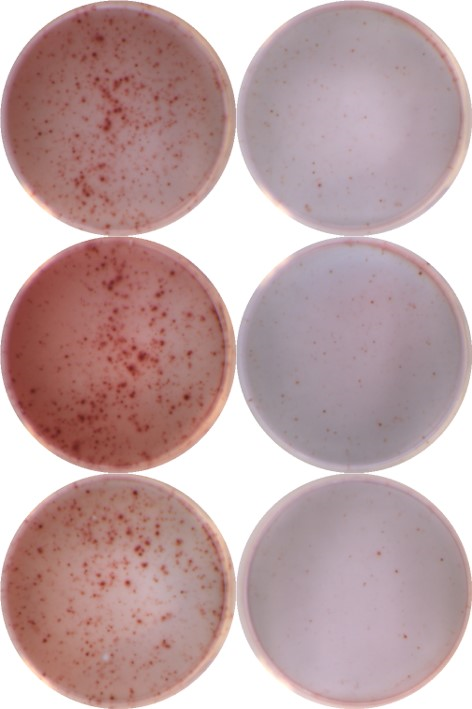

Supplement: Supplementary file 4 — Source data Fig. 2 [file 44321_2024_120_MOESM4_ESM.zip › Figure2 Source Data/Fig. 2D/Fig. 2D.tif]

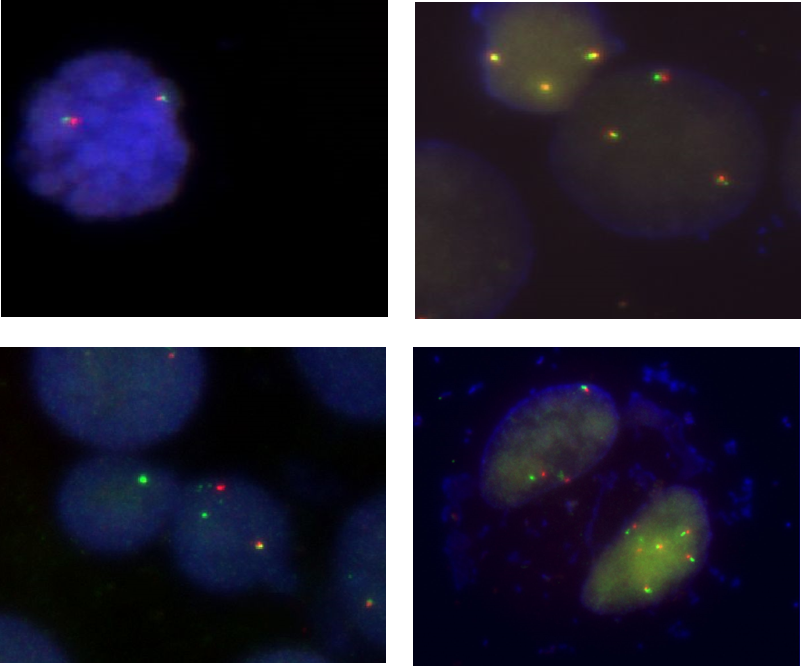

Supplement: Supplementary file 5 — Source data Fig. 3 [file 44321_2024_120_MOESM5_ESM.zip › Figure3 Source Data/Fig. 3A/Fig. 3A.tif]

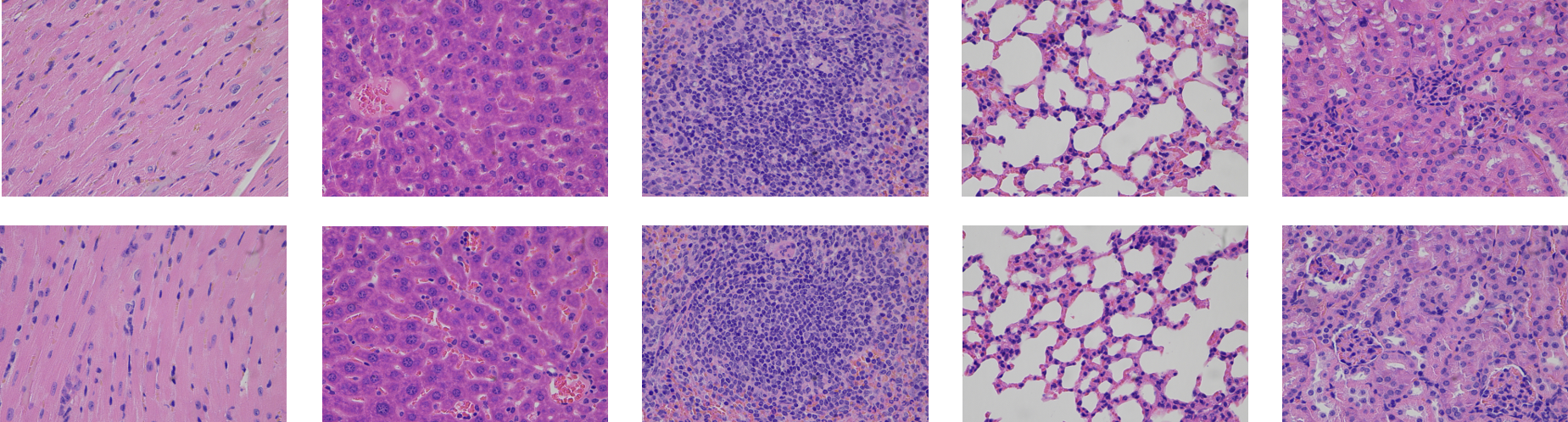

Supplement: Supplementary file 5 — Source data Fig. 3 [file 44321_2024_120_MOESM5_ESM.zip › Figure3 Source Data/Fig. 3H/Fig. 3H.tif]

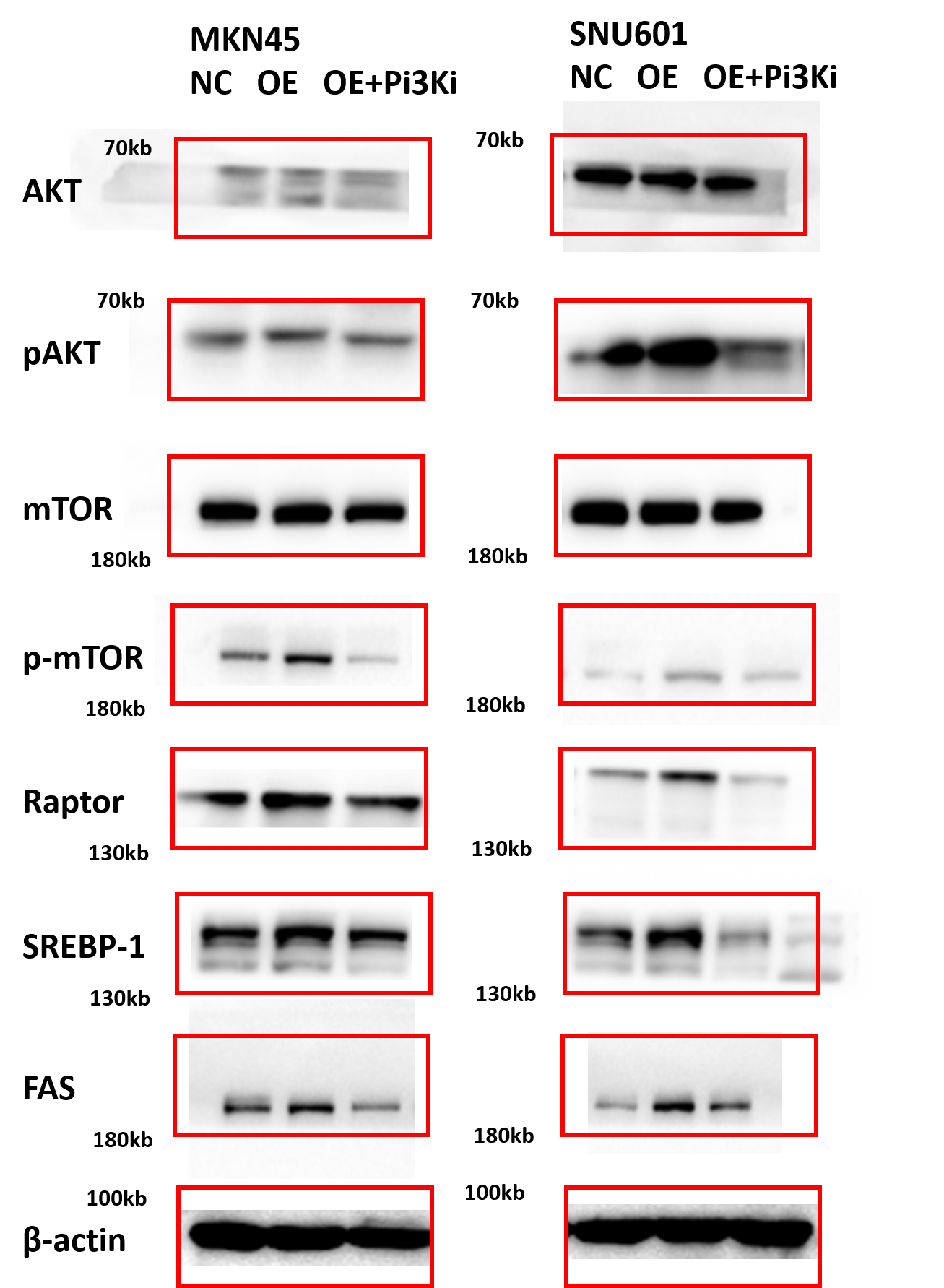

Supplement: Supplementary file 7 — Source data Fig. 5 [file 44321_2024_120_MOESM7_ESM.zip › Figure5 Source Data/Fig. 5F/Fig. 5F.tif]
